# Combined Transcriptomic and Metabolomic Analysis of the Coloration Mechanism in Colored-Leaf *Osmanthus fragrans* ‘Jinyu Guihua’

**DOI:** 10.3390/plants15030385

**Published:** 2026-01-27

**Authors:** Peng Guo, Yu Huang, Peiquan Jin, Xinke Li, Qianqian Ma, Luoyi Yu, Wei Zhao, Yihan Wang, Fude Shang

**Affiliations:** 1 College of Life Sciences, Henan Agricultural University, Zhengzhou 450046, China; 6253730@163.com (P.G.); 18079089659@163.com (Y.H.); 18437987793@163.com (P.J.); 15236728906@163.com (X.L.); 15036040308@163.com (Q.M.); y2997158519@163.com (L.Y.); zhaowei19991023@163.com (W.Z.); 2 Henan *Osmanthus* Germplasm Innovation and Resource Utilization Engineering Research Center, Zhengzhou 450046, China

**Keywords:** ‘Jinyu Guihua’, RNA-seq, metabolome, leaf color mutant, chloroplasts

## Abstract

The colored-leaf *Osmanthus fragrans* is a valuable ornamental tree species that integrates greenery, colorful leaves, and fragrance. At present, research on colored-leaf *Osmanthus fragrans* mainly focuses on cultivar breeding, classification and cultivation, and physiological resistance, while studies on leaf color variation remain limited. In this study, the colored-leaf *Osmanthus* cultivar ‘Jinyu Guihua’ and its female parent were used as materials. The leaf coloration mechanism was systematically investigated through a combined analysis of physiology, transcriptomics, and metabolomics. The results showed that compared with the female parent, the leaves of ‘Jinyu Guihua’ exhibited significantly reduced chlorophyll b and anthocyanin contents, fewer chloroplasts, and more plastoglobules. Transcriptomic analysis identified 3938 differentially expressed genes (DEGs), among which the key chlorophyll metabolism gene *CAO* was downregulated and *NOL* was upregulated; the key carotenoid synthesis gene *PSY* was downregulated and *CYP97A3* was upregulated; the key anthocyanin synthesis gene *ANS* was downregulated; and the *PetC2* gene in the photosynthesis-related Cytb6-f complex was upregulated. qRT-PCR validation results were consistent with the RNA-seq data. Metabolomic analysis detected 1290 metabolites, classified into 21 subcategories, with flavonoids being the most abundant (17.21%). Anthocyanin synthase (*ANS*) significantly downregulated the expression levels of cyanidin-3-O-rutinoside (Cy3R) and delphinidin-3-O-rutinoside (De3R). In conclusion, the leaf color variation in ‘Jinyu Guihua’ is closely related to changes in leaf pigment content and the regulation of key metabolic pathway gene expression. The findings of this study provide a theoretical basis for the molecular breeding of new colored-leaf *Osmanthus* varieties and serve as a reference for trait research in other ornamental plants.

## 1. Introduction

*Osmanthus fragrans* is a plant belonging to the Oleaceae family that holds significant ornamental, nutritional, and medicinal value [1,2]. It appears in five varieties based on flower color, flowering period, and leaf color: *Osmanthus fragrans* (Luteus Group), *Osmanthus fragrans* (Albus Group), *Osmanthus fragrans* (Aurantiacus Group), *Osmanthus fragrans* (Siji Group), and *Osmanthus fragrans* (Color Group) [3]. The colored-leaf *Osmanthus fragrans* variety group (established in 2014) evolved from other varieties of *Osmanthus fragrans* as a result of both long-term natural selection and artificial selection. *Osmanthus fragrans* is known traditionally for its pleasant scent. The colored-leaf variety also holds great ornamental value. Owing to its greening, aromatization, and colorization, it has broad applications in botany [4]. There are 57 known varieties of colored-leaf *Osmanthus fragrans* [5]. These are divided into two categories according to their different color periods, namely, perennial and seasonal. For example, the ‘Yuntian Caigui’ variety maintains the same leaf color throughout the year [6]. The spring, summer, and autumn shoots of the ‘Yongfu Caixia’ variety have colored leaves at germination, while new leaves that flush after the shoots have sealed are green, resembling the established leaves of 2–3 years old. The ornamental period of colored-leaf *Osmanthus fragrans* is from March to July and from September to November [7]. Although studies have been conducted on the mechanism of its seasonal leaf coloring [8], the molecular mechanism of its perennial leaf coloring remains unclear.

The appearance of colored leaves is mainly caused by structural variations in chloroplasts, changes in pigment content, and abnormal pigment-related gene pathway synthesis and degradation [9]. Compared to the structural characteristics of normal green leaves, colored leaves exhibit an abnormal microstructure and ultrastructure [10,11]. Chloroplasts are important organelles for plant photosynthesis. A lack of chlorophyll b in plants leads to incomplete chloroplast development, incomplete ultrastructure, thylakoid damage, and the aggregation and proliferation of plastids [12]. Studies have shown that a decrease in the number of chloroplasts inhibits the expression of genes related to the light-harvesting complex (LHC), resulting in the inhibition of chlorophyll synthesis and an increase in the Chl-a/Chl-b ratio [13]. In *Eucommia ulmoides*, the contents of Chl-a, Chl-b, and total chlorophyll in red leaves are significantly lower than those in green leaves [14]. *PetC* is a subunit protein involved in photosynthesis. It is encoded by a nuclear *PetC* gene, which is downregulated in the *Forsythia* ‘Gold Leaf’ mutant [15]. Additionally, the chloroplasts in the green areas of orchid leaves (‘Dharma’ variety) have different degrees of developmental defects, while the chloroplasts in the yellow areas are severely degraded, with an incomplete chloroplast structure [16]. Similarly, studies on chlorophyll-deficient mutants in flue-cured tobacco have revealed that the albino mutant (Al) exhibits disordered cellular structure, a reduced number of chloroplasts, and an absence of starch grains. The light-green mutant (SG), while structurally normal, shows significantly smaller chloroplast and starch grain areas compared to the wild type [17].

Leaf color mutations mainly relate to changes in chlorophyll content in the chloroplast. Chl-a is associated with a blue-green color, and Chl-b with yellow-green [18]. The chlorophyll cycle involves a mutual transformation of Chl-a and Chl-b, whereby Chl-b is first synthesized by Chl-a and then catabolized after Chl-a transforms into Chl-b. Genes related to the chlorophyll cycle play an important role in photosynthesis [12]. In etiolated and yellow-green mutants, Chl-b generally decreases more markedly than Chl-a, which increases the Chl-a/Chl-b ratio accordingly [19]. If the gene mutation occurs during the early stage of chlorophyll synthesis, the leaves usually exhibit yellowing and albinism. If the gene mutation occurs during the late stage, the leaves usually exhibit patches and stripes [20]. The *NOL* gene encodes Chl-b reductase and catalyzes the conversion of Chl-b to Chl-a, and its overexpression influences the accumulation of chlorophyll [21]. This phenomenon is exemplified in the yellow leaves of chlorophyll-deficient tea plants, where the transcription level of the *CsCAO* gene is increased and the Chl-b content is decreased [22]. Similarly, in the colored-leaf mutant of *Cymbidium hybridum*, the chlorophyll content is decreased, and the expression of key genes such as *CLH*, *PAO*, and *NOL* is increased [23].

Carotenoids can cause plant leaves to turn orange or yellow and play an important role in plant photosynthesis. Carotenoids include capsanthin (red), β-carotene (orange-red), lycopene (pink), violaxanthin (orange-yellow), lutein (yellow), and zeaxanthin (yellow) [24]. When chlorophyll content is greatly reduced and carotenoid content is increased, leaves appear more yellow [25]. For example, in the yellow leaves of *Oncidium*, the downregulation of *PSY* disrupts the carotenoid biosynthesis pathway, ultimately leading to leaf yellowing [26].

The color transition of leaves from green to yellow or red is primarily attributable to the accumulation of anthocyanins and changes in the proportions of other pigments [27]. In *Acer palmatum* leaves, for instance, increased anthocyanin content induces red coloration [28]; in the *Paeonia suffruticosa* (‘Manyuan Chunse’ variety), elevated anthocyanin levels coupled with reduced chlorophyll and carotenoid content collectively contribute to red leaf pigmentation [29]; during the development of red maple leaves, anthocyanin content significantly decreases as the leaf color changes from red to green, while chlorophyll content substantially increases [30].

Some transcription factor families can also regulate pigment-related genes, such as NAC25, which affects the expression of *PDS*, *PSY,* and other genes, as well as the accumulation of carotenoids [31]. MYB, BHLH, and WD40 control the expression of structural genes such as *ANS* in the anthocyanin synthesis pathway [32]. AP2/ERF is involved in the regulation of plant aroma and coloration [33]; WRKY6 and DELLA can regulate senescence and chlorophyll degradation [34].

The complex biological mechanisms of various plants have been studied frequently in recent years. High-throughput sequencing technology has matured, and omics have become an important method for studying plant coloration. Joint analyses of plant transcriptomics and metabolomics can not only predict metabolite changes at the transcriptional level, but also verify gene transcription results at the metabolic level. These can be mutually verified using omics. The addition of physiological experimentation could provide a more comprehensive and convenient approach to analyzing the complex processes behind leaf coloration [35,36,37,38].

Multiomics research and analysis have been conducted to investigate the leaf coloration of many plants, including the *banyan tree* [39], *red sandalwood* [40], *crape myrtle* [41], *birch* [42], and *peony* [43]. Studies on orchid mutants (‘Dharma’ variety) have shown that genes encoding key chlorophyll degradation enzymes are highly expressed, and that changes in leaf color may be due to the excessive degradation of chlorophyll [44]. In the yellow-green leaf mutant of birch, the ratio of Chl-a to Chl-b is relatively high [42]. Yang et al. conducted transcriptome sequencing analysis of both wild type and leaf color mutants of *Anthurium andraeanum*, and found that chlorophyll biosynthesis is blocked in young, mature, and old leaves of leaf color mutants due to changes in chlorophyll and intermediate metabolite content [45]. Lu et al. found that color changes in maple safflower during autumn are due to the upregulated expression of a large number of genes in the anthocyanin synthesis pathway and increases in cyanidin 3-(6″-acetyl-galactoside) and cyanidin 3-arabinoside content [46].

The colored-leaf Osmanthus fragrans ‘Jinyu Guihua’ originated from a bud mutation of its female parent. In this study, ‘Jinyu Guihua’ and its female parent were comparatively analyzed to elucidate the physiological, structural, and molecular mechanisms underlying leaf coloration. Microscopic and ultrastructural observations, pigment content determination, transcriptomic analysis, and metabolomic profiling were integrated to identify key genes and metabolites associated with leaf color formation. The coloration mechanism of ‘Jinyu Guihua’ was extensively analyzed to provide a theoretical basis for further research on the improvement and application of colored-leaf *Osmanthus fragrans* varieties.

## 2. Results

### 2.1. Physiological Experimental Results

#### 2.1.1. Leaf Microstructure Observations

**Figure 1:**
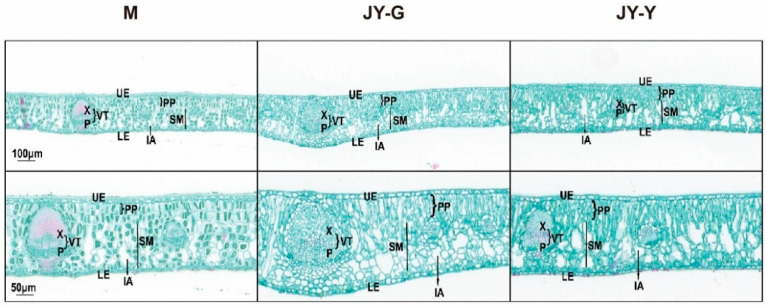
Paraffin section results (Top to bottom: 100 μm in the first row; 2nd row 50 μm). Note: X: xylem, P: phloem, VT: vascular tissues, UE: upper epidermis, LE: lower epidermis, PP: palisade parenchyma, IA: intercellular air space, SM: spongy mesophyll.

Microstructural observations of the paraffin-sectioned cross-sections revealed that the palisade and spongy tissue cells of the female parent’s leaves were regular and close, with a large number of chloroplasts and gaps between the mesophyll cells. Compared to the female parent leaves, there were fewer chloroplasts in the green part of the ‘Jinyu Guihua’ leaves, with thicker palisade tissue, more loosely arranged sponge tissue, and larger gaps between the mesophyll cells. In the yellow part, the cells were irregularly long, with even fewer chloroplasts, thicker palisade tissue, more loosely arranged sponge tissue, and larger intercellular spaces (Figure 1).

#### 2.1.2. Leaf Ultrastructure Observations

The ultrastructure results showed that the cell shape of the female parent’s (M) leaves was relatively regular, the cell membrane was not obviously damaged, the cell wall was intact and continuous, with uniform thickness and no obvious plasmolysis. The number of chloroplasts was abundant; no obvious swelling was observed, the membrane was complete, the lamellar structure of the grana thylakoid was clear, and no obvious expansion of thylakoids was observed in the matrix. A small number of plastid balls were observed with uneven sizes and a uniform dense core. There was no obvious swelling of the mitochondria, the double membrane was intact, and the matrix was uniform. There was no observable damage to the vacuole membrane.

**Figure 2:**
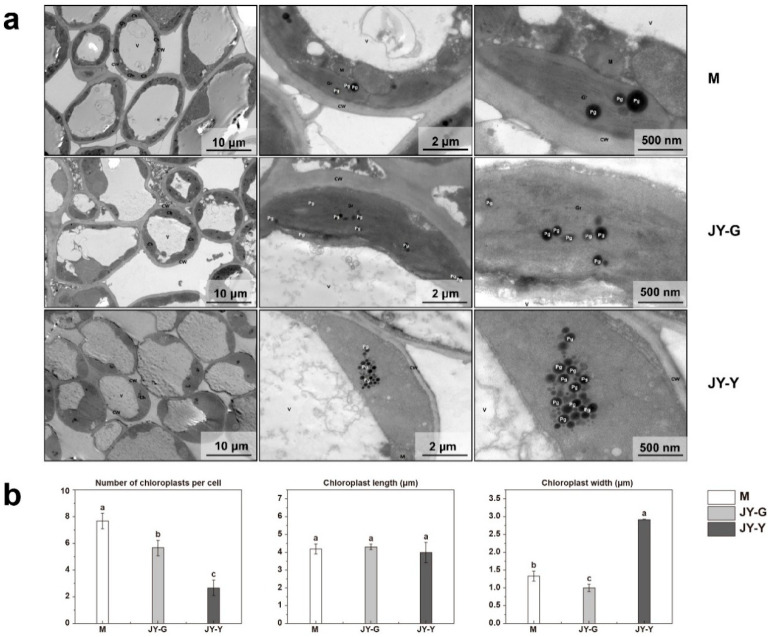
(**a**) Ultrastructure of mesophyll chloroplasts observed by transmission electron microscopy. (**b**) Differences in the number and size of chloroplasts among samples. Note: (**a**) CW: Cell wall; Ch: Chloroplast; V: vesicles; Pg: plasma globules, M: mitochondrial, Gr: basal granule. Vesicles (**b**) Statistical significance was assessed using one-way ANOVA, and different lowercase letters marked on data bars indicate significant differences (*p* < 0.05).

Compared to the female parent, the cell shape of the green part (JY-G) of the ‘Jinyu Guihua’ leaves was more regular. The cell membrane was not obviously damaged, and the cell wall was intact and continuous with a uniform thickness and no obvious plasmolysis. The chloroplast count was moderate, and no obvious swelling was observed. The membrane was intact, the grana thylakoid lamellar structure was present, and no obvious expansion was observed in the stroma. The number of plastid balls was regular, with uniform sizes and a uniform dense core. Free organelles were observed within the vacuolar membrane. The cell shape of the yellow part (JY-Y) of the ‘Jinyu Guihua’ leaves was more regular. The cell membrane was not obviously damaged, and the cell wall was intact and continuous with a uniform thickness and no obvious plasmolysis. The chloroplasts were slightly swollen with a uniform matrix, vacuolar degeneration of the organelles, no obvious grana lamellar structure, clear matrix thylakoid expansion, and vacuolar degeneration. A large number of plastid balls were observed with an irregular shape, a uniform dense core, and local aggregation. The mitochondria were slightly swollen, the membrane was intact, and a small area of the matrix was shallow and dissolved. There was no obvious damage to the vacuolar membrane (Figure 2a).

In addition, the intracellular chloroplasts of all three groups of leaf cells (M, JY-G, and JY-Y) were randomly measured. The number of chloroplasts decreased along a gradient: the M sample had the highest chloroplast count, followed by the JY-G and JY-Y samples. There was no significant difference in the length of the chloroplasts. The JY-Y sample had a significantly larger chloroplast width compared to the M sample, and the JY-G sample had the smallest chloroplast width (Figure 2b).

#### 2.1.3. Pigment Content Determination

The results of leaf pigment content measurements showed that the chlorophyll a content in sample JY-G was significantly higher than that in sample M, whereas the chlorophyll a content in sample JY-Y was lower than that in sample M. Compared with sample M, the chlorophyll b contents of both JY-G and JY-Y were significantly reduced, with JY-Y being significantly lower than JY-G. In terms of total chlorophyll content (the sum of chlorophyll a and chlorophyll b), sample JY-G was slightly higher than sample M, while sample JY-Y was significantly lower than sample M.

**Figure 3:**
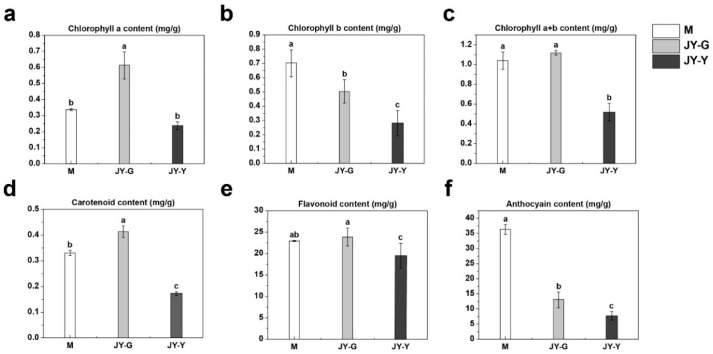
Results of pigment determination showing the contents of chlorophyll a (**a**), chlorophyll b (**b**), total chlorophyll (a + b) (**c**), carotenoids (**d**), flavonoids (**e**), and anthocyanins (**f**) Note: Statistical significance was assessed using one-way ANOVA, and different lowercase letters marked on data bars indicate significant differences (*p* < 0.05).

With respect to other pigment components, the carotenoid content in sample JY-G was significantly higher than that in sample M, whereas it was significantly lower in sample JY-Y than in sample M. The flavonoid content in sample JY-G was slightly higher than that in sample M, while sample JY-Y showed a significant reduction compared with sample M. Regarding anthocyanin content, both JY-G and JY-Y were significantly lower than sample M, with JY-Y being significantly lower than JY-G (Figure 3).

### 2.2. Transcriptomic Results and Analysis

**Figure 4:**
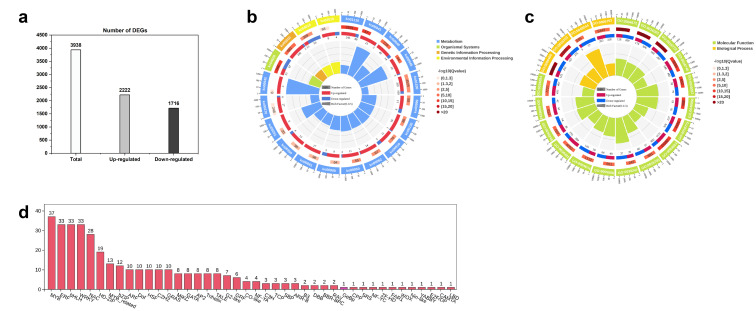
(**a**) DEG histogram, (**b**) GO enrichment circle plots of DEGs, (**c**) KEGG enrichment circle plots of DEGs, (**d**) Screening of transcription factors in differential gene categories.

Using (| log2 FC | > 1 and FDR < 0.05) as the standard, 3938 differentially expressed genes (DEGs) were screened. Among these, 2222 genes were upregulated and 1716 were downregulated (Figure 4a). Based on the Q values, the 20 pathways with the most significant GO and KEGG enrichment were selected to draw the GO/KEGG circle diagram. The differential gene enrichment map shows that the 20 pathways with the highest level of GO enrichment included protein kinase activity (GO: 0004672), protein phosphorylation (GO: 0006468), phosphorylation (GO: 0016310), heme binding (GO: 0020037), polysaccharide binding (GO: 0030247), oxidoreductase activity (GO: 0016491) and so on (Figure 4b). The 20 pathways with the highest levels of KEGG enrichment included secondary metabolite biosynthesis (ko01110), flavonoid biosynthesis (ko00941), phenylpropanoid biosynthesis (ko00940), plant secondary metabolite biosynthesis (ko00999), anthocyanin biosynthesis (ko00942), etc. (Figure 4c).

Transcription factor (TF) gene annotation was performed using the Plant Transcription Factor Database (https://planttfdb.gao-lab.org/, accessed on 16 November 2025) to determine whether the genes were members of a TF family and to summarize how many genes belonged to each TF family. The screened differential genes were annotated, and 351 TFs were screened. The genes belonged to 42 different TF families. Among the differential TFs, the number of MYB TF families accounted for the largest proportion (37, accounting for 10.54%), followed by ERF (33, accounting for 9.40%), bHLH (33, accounting for 9.40%), WRKY (33, accounting for 9.40%), and NAC (28, accounting for 7.98%) (Figure 4d).

### 2.3. Metabolomic Results and Analysis

**Figure 5:**
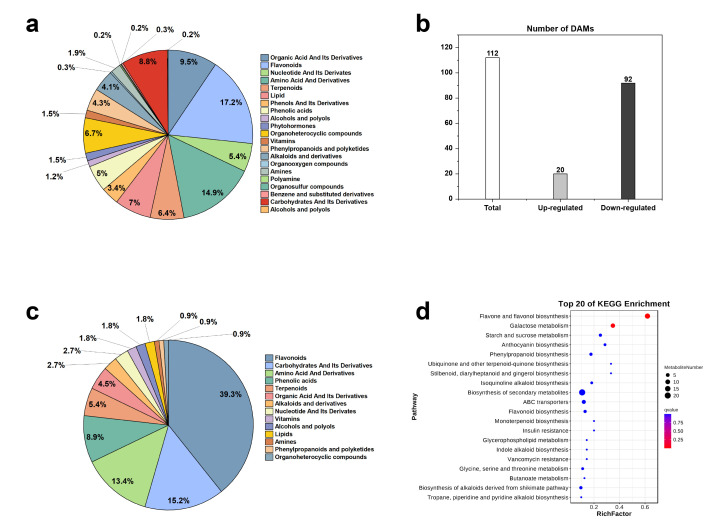
(**a**) Metabolite chart (**b**), DAM charts (**c**), DAM metabolite chart (**d**), KEGG enrichment bubble map.

A total of 1290 differential metabolites were detected and divided into 21 subcategories. These included 222 flavonoids, 192 amino acids and their derivatives, 122 organic acids and their derivatives, 90 lipids, 87 organic heterocyclic compounds, etc. (Figure 5a). A total of 112 differential metabolites were screened, of which 20 were upregulated and 92 were downregulated (Figure 5b). These were divided into 14 categories, including 44 flavonoids, 17 sugars and their derivatives, and 15 amino acids and their derivatives (Figure 5c). The KEGG database was used to conduct differential metabolite enrichment analysis, and the KEGG enrichment bubble diagram was drawn accordingly, with the first 20 pathways having the lowest Q values (Figure 5d). The differential metabolites were significantly enriched in the flavonoid and flavanol biosynthesis pathway (ko00944), the galactose metabolism pathway (ko00052), the anthocyanin biosynthesis pathway (ko00942), the phenylpropanoid biosynthesis pathway (ko00940), the flavonoid biosynthesis pathway (ko00941), and other metabolic pathways.

### 2.4. qRT-PCR Identification

**Figure 6:**
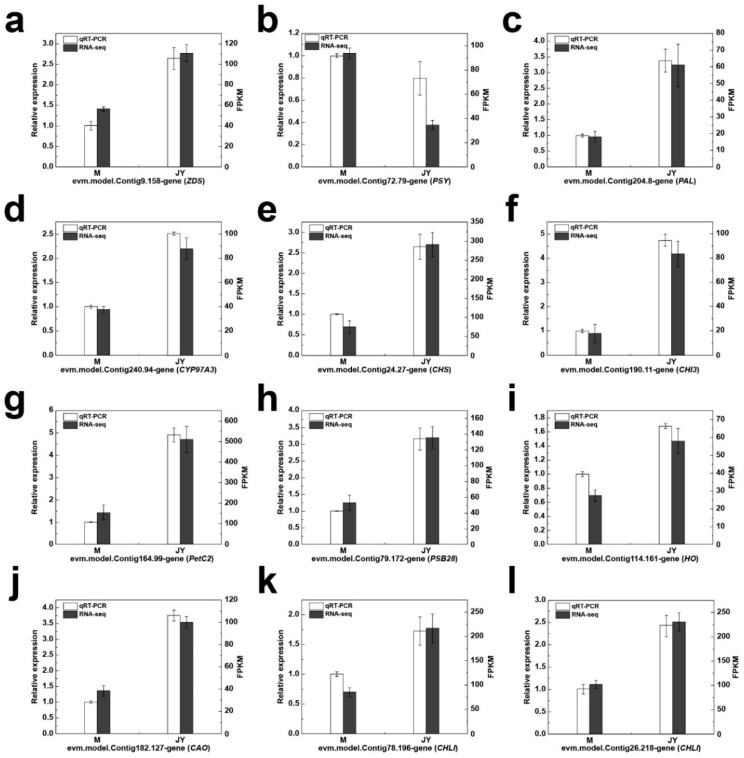
qRT-PCR verification of differentially expressed genes (DEGs). Subfigures represent different genes: (**a**) evm.model.Contig9.158-gene (ZDS), (**b**) evm.model.Contig72.79-gene (PSY), (**c**) evm.model.Contig204.8-gene (PAL), (**d**) evm.model.Contig240.94-gene (CYP97A3), (**e**) evm.model.Contig24.27-gene (CHS), (**f**) evm.model.Contig190.11-gene (CHI3), (**g**) evm.model.Contig164.99-gene (PetC2), (**h**) evm.model.Contig79.172-gene (PSB28), (**i**) evm.model.Contig114.161-gene (HO), (**j**) evm.model.Contig182.127-gene (CAO), (**k**) evm.model.Contig78.196-gene (CHLI), and (**l**) evm.model.Contig26.218-gene (CHLI). Note: the value of qRT-PCR responds in the form of relative expression quantity, and the value of RNA-seq responds in the form of FPKM value.

To study the levels of expression of differential genes related to photosynthesis, chlorophyll metabolism, carotenoid metabolism, and anthocyanin metabolism, 12 key differential genes were selected. Their expression patterns were measured in the leaves of the female parent plant and ‘Jinyu Guihua’ via qRT-PCR. Four of these genes were related to the chlorophyll pathway, three to the carotenoid metabolism pathway, three to the flavonoid metabolism pathway, and the remaining two to the photosynthesis pathway. The qRT-PCR expression trends of these 12 key differential genes were fully consistent with the transcriptomic data, indicating that the RNA-seq data were stable and reliable (Figure 6).

## 3. Discussion

### 3.1. The Effects of the Photosynthesis Pathway on Leaf Coloration in ‘Jinyu Guihua’

In photosynthetic photoreactions, the four major protein complexes involved in the electron transport chain are Photosystem II (PSII), the cytochrome b6f complex, Photosystem I (PSI), and the F-type H^+^-ATP synthase complex. The accumulation of the PSI and PSII supercomplexes (including the light-harvesting antenna complex) is abnormal in *Arabidopsis* evr3-1/egy1 mutants, resulting in a ‘spotted-leaf’ color phenotype [47]. In the golden-leaved mutant of *Forsythia suspensa*, the expression of the *PetC* gene is downregulated and unaffected by light intensity [15].

**Figure 7:**
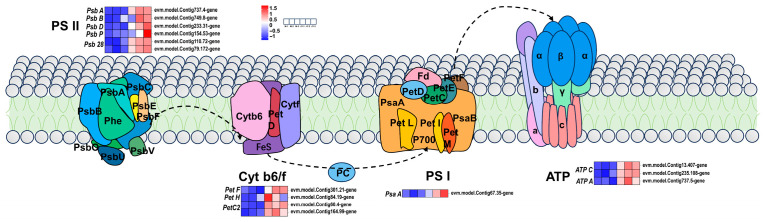
Differential gene expression patterns related to photosynthesis. Note: Scale bars indicate gene expression patterns (Red: Up, Blue: Down, White: No change, Normalized Signal Intensity: −1.0 to 1.5, consistent with the color change from blue to red). Cyt b6f: cytochrome b6f complex, FD: ferredoxin, PC: plastocyanin, PSI: Light System I; PSII: Photosystem II, PsaA: photosystem I P700 chlorophyll a apoprotein A1, PsaB: photosystem I P700 chlorophyll a apoprotein A2, PC: plastocyanin, beta: F-type H+ transporter ATPase subunit β.

In this study, we further analyzed the expression patterns of genes related to the photosynthesis pathway. The differential genes *psbA*, *psbB*, *psbD*, and *psb28* were found to be related to photosynthesis. The differential genes *petH*, *petC2*, and *petF* are related to the cytochrome b6/f complex. The differential genes *ATPA* and *ATPC* (which encode ATPase), the differential gene *psaA*, and the PSI encoding system were all upregulated in the leaves of ‘Jinyu Guihua’. Among them, six differential genes in the PSII system related to the Psb gene family (*psbA*, *psbB*, *psbD*, and *psb28*) were upregulated. The pet gene family (encoding the cytochrome b6/f complex) and proteins related to the photosynthetic electron transport process contained four DEGs, namely, *petH* (encoding NADP+ oxidoreductase), *petC2* (encoding the cytochrome b6-f complex iron sulfur subunit), and *petF* (encoding oxidoreductase). Three DEGs encoding F-type ATPase and the F-type H+/Na+ transport ATPase-alpha subunit (*ATPA*), and two ATPC genes were upregulated. The differential gene *psaA* (belonging to the psa gene family) encodes the PSI system, and these subunit proteins are mainly involved in the composition of the PSI reaction center (Figure 7).

Microstructural characterizations of the leaf samples in this study show that ‘Jinyu Guihua’ has certain structural abnormalities. Compared with the female parent plant, *Osmanthus fragrans*, the morphological differences in the palisade and spongy tissues and the number of chloroplasts in ‘Jinyu Guihua’ (Figure 7) are similar to the anatomical differences between the bright and green areas of *crabapple* and *banyan* leaves [48]. Other studies have shown that these results are related to the lack of any significant reduction in chloroplasts or photosynthetic capacity in the leaf mottling mechanism of *B. rex* [49]. Ultrastructural observations of chloroplasts in the highly light-sensitive zebra leaf mutant, TCM248, showed that the chloroplast structure in the green leaf areas was normal, while the chloroplast lamellar system in the yellow areas was visibly disordered. The excessive light absorption of plant photosynthetic pigments can lead to the destruction of chloroplast structure due to photooxidation [50].

In summary, the low chlorophyll content in ‘Jinyu Guihua’ may promote the production of photosynthetic enzymes to maintain normal growth. The DEGs of PSI, PSII, and Cytb6-f components were upregulated in ‘Jinyu Guihua’ leaves. Notably, the log2FC values of *PetC2* were 1.71 and 5.44, respectively, showing their significant differentiation. This also aligns with the pigment determination results. Therefore, we speculated that the upregulated expression of genes related to the photosynthesis pathway in ‘Jinyu Guihua’ may represent a compensatory response to decreased chlorophyll content, contributing to the maintenance of normal growth. This compensatory photosynthetic regulation highlights an adaptive strategy of Osmanthus fragrans leaf color mutants, indicating that ‘Jinyu Guihua’ can sustain photosynthetic efficiency under reduced chlorophyll conditions. Such a mechanism provides a physiological basis for the stable growth and horticultural utilization of yellow-leaf cultivars in Osmanthus fragrans breeding and landscape applications.

### 3.2. The Effects of Chlorophyll Metabolism on Leaf Coloration in ‘Jinyu Guihua’

There are two main types of chlorophylls, namely, chlorophyll a and chlorophyll b. Chlorophyll a is the primary pigment. During photosynthesis, light energy is converted into chemical energy through several steps. The photosynthetic capacity of plants is largely determined by the relative chlorophyll a content [51]. The chlorophyll metabolism pathways include the synthesis of chlorophyll a, the chlorophyll cycle, and chlorophyll degradation.

In this study, pigment determinations revealed that the chlorophyll a, chlorophyll b, and total chlorophyll content of the yellow part of ‘Jinyu Guihua’ leaves were significantly lower than in the green part and in the female parent’s leaves. Chlorophyll a was significantly higher in the green part than in the female parent’s leaves, chlorophyll b was lower than in the female parent’s leaves, and the total chlorophyll content did not differ significantly from that of the female parent’s leaves. This indicates that the change in chlorophyll content in ‘Jinyu Guihua’ leaves affects the phenotype at the physiological level.

*Chlorophyllide a oxygenase (CAO)* is a key regulatory enzyme responsible for the synthesis of chlorophyll b, which independently catalyzes two consecutive oxidation reactions to convert chlorophyllide a into chlorophyllide b [52]. The overexpression of *AtCAO* in chlorophyll-b-deficient *Arabidopsis* leaves can cause the accumulation of large amounts of chlorophyll b [53]. Lee et al. also confirmed that the abnormal expression of *OsCAO*1 can affect chlorophyll b synthesis in rice, resulting in a phenotype with pale green leaves, slowed growth rates, and fewer tillers [54].

**Figure 8:**
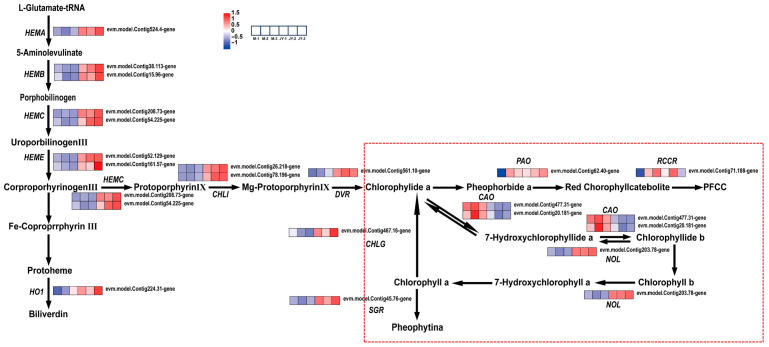
Differential gene expression patterns related to chlorophyll synthesis and degradation. Note: The scale bar represents gene expression patterns (red: up, blue: down, white: no change, normalized signal intensity: −1.0 to 1.5, consistent with the color change from blue to red); Red dotted line: chlorophyll degradation pathway.

In this study, 13 genes involved in chlorophyll biosynthesis and modification, as well as 10 chlorophyll degradation-related enzymes, were identified through KEGG pathway analysis. Among these, the genes regulating chlorophyll a and chlorophyll b enzymes in the chlorophyll degradation pathway were significantly altered (Figure 8). The expression of chlorophyll oxygenase (*CAO*) in the leaves of ‘Jinyu Guihua’ was downregulated, which affects the conversion of chlorophyll a to chlorophyll b. The upregulation of chlorophyll b reductase (*NOL*)—which is responsible for catalyzing the degradation of chlorophyll b and maintaining the stability of the photosystem—accelerates the degradation of chlorophyll. This supports the results of previous physiological experiments on pigment content. In addition, qRT-PCR further confirmed the altered expression of *NOL* and *CAO* in the leaves of ‘Jinyu Guihua’.

In summary, pigment determination confirmed that chlorophyll a, chlorophyll b, and total chlorophyll content were significantly decreased in the yellow part of ‘Jinyu Guihua’ leaves, while the chlorophyll a content in the green part was significantly increased. In terms of chlorophyll metabolism, the downregulation of the *CAO* gene and the upregulation of the *NOL* gene accelerate the chlorophyll degradation rate and decrease chlorophyll accumulation in the leaves of ‘Jinyu Guihua’. The decreased chlorophyll b content observed mainly in the yellow leaf sectors may explain the leaf color variation in ‘Jinyu Guihua’. These findings indicate that the regulation of chlorophyll metabolism is a key determinant of leaf color formation in Osmanthus fragrans, and provide a molecular basis for manipulating yellow–green leaf traits in ornamental germplasm improvement.

### 3.3. The Effects of Carotenoid Metabolism on Leaf Coloration in ‘Jinyu Guihua’

Carotenoids are a class of natural pigments that are widely distributed in nature and play an important role in determining the ornamental value of plants. The carotenoid metabolic pathway controls the formation of yellow and orange colors in plants, and affects the synthesis of volatile aromatic compounds, thereby affecting pigment deposition and aroma production in the organs of ornamental plants [55]. Carotenoids include α-carotene, β-carotene, lycopene, and its oxygenated derivatives lutein, zeaxanthin, and violaxanthin [56]. β-carotene hydroxylase in the carotenoid metabolic pathway catalyzes β-carotene, which increases zeaxanthin content in plant cells [57]. *LUT5* is responsible for encoding carotenoid hydroxylase *CYP97A3*, which supports carotenoid hydroxylation and plays a positive regulatory role in the carotenoid pathway, thereby promoting the formation of carotenoids [58]. The overexpression of *CYP97A3* significantly reduces α-carotene content in the leaves and roots of orange carrots, decreasing the total carotenoid content by between 30 and 50 amount of upstream *PSY* proteins also decreases accordingly, indicating that *CYP97A3* has a reverse negative regulatory effect on the level of *PSY* protein and controls the conversion of carotenoids to the α- and β-branches, thereby affecting the content and ratio of α-carotene and β-carotene in carrots [59]. The yellowing of hybrid orchid leaves indicates a downregulation of *PSY*, resulting in changes in metabolic pathways that encode carotenoids, gibberellin, and abscisic acid, as well as chlorophyll biosynthesis [26]. Violaxanthin decyclase enzyme (*VDE*) is related to the synthesis of orange-yellow violaxanthin. An increase in violaxanthin content promotes the yellowing of plant leaves and fruits. For example, the fruits of the ‘*Red Summer Orange*’ citrus plant accumulate high levels of violaxanthin and are therefore yellow in color [60]. *VDE* and *LUT5* are significantly upregulated in *pepper* leaves, while *ZEP* is significantly downregulated. This expression trend promotes significant increases in the zeaxanthin and violaxanthin content in mutants under high light conditions, resulting in the yellowing of leaves [61].

In this study, carotenoids first formed phytoene (colorless, with three conjugated double bonds and a C40 carotenoid skeleton) via GGPP (geranylgeranyl pyrophosphate), before gradually desaturating through 9,15,9′-tricis-ε-carotene and 9,9′-dicis-ε-carotene to form lycopene, which then produced α-carotene and β-carotene. However, the expression of *LUT2* was decreased. Since *LUT2* has a positive regulatory effect on α-carotene, this resulted in the transformation of carotenoid synthesis from the α-branch to the β-branch in the leaves of ‘Jinyu Guihua’. Therefore, this conversion to the β-branch may affect carotenoid content and thus affect the leaf color of ‘Jinyu Guihua’.

**Figure 9:**
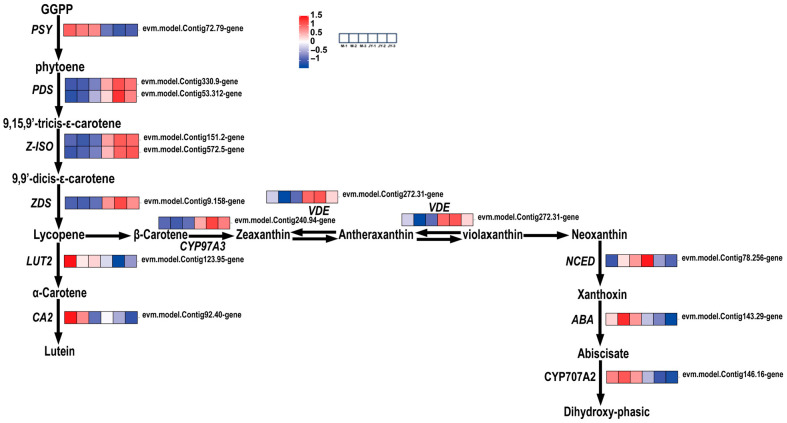
Analysis of differential gene expression related to carotenoid biosynthesis. Note: the scale bar represents gene expression patterns (red: up, blue: down, white: no change, normalized signal intensity: −1.0 to 1.5, consistent with the color change from blue to red).

In summary, a total of 14 genes were found to be differentially expressed and significantly altered in the carotenoid pathway of ‘Jinyu Guihua’, compared to its female parent. The downregulation of *LUT2* may lead to increased conversion to the β-carotene branch, while the downregulation of *CYP97A3* plays a negative regulatory role in *PSY*, causing the upregulated *VDE* gene to promote the formation of yellow zeaxanthin (Figure 9). This affects the proportion of pigments in the leaves of the ‘Jinyu Guihua’ phenotype, affecting its leaf coloration. In Osmanthus fragrans, these carotenoid pathway alterations help explain the enhanced yellow pigmentation of ‘Jinyu Guihua’ leaves and identify potential molecular targets for increasing leaf color diversity and ornamental value through metabolic regulation.

### 3.4. The Effect of Anthocyanin Metabolism on Leaf Coloration in ‘Jinyu Guihua’

Anthocyanins are flavonoid compounds that promote red, purple, and blue coloration [62]. The synthesis of anthocyanins in plants is strongly influenced by the structural genes of the metabolic pathways, such as phenylalanine ammonia lyase (*PAL*), chalcone synthase (*CHS*), flavanone 3-hydroxylase (*F3H*), dihydroflavonol 4-reductase (*DFR*), and anthocyanin synthase (*ANS*) [63]. During the development of purple buds in the tea cultivar ‘Wuyiqizhong 18′, the contents of key enzymes involved in the flavonoid and anthocyanin biosynthesis pathways, including chalcone synthase (*CHS*), chalcone isomerase (*CHI*), and flavonol synthase (*FLS*), are significantly higher in tender purple leaves than in mature green leaves [64]. When the *ANS* gene is mutated in *Paeonia suffruticosa*, leaf color shifts from purple to green [29]. While studying the spotted and green leaves of the colored-leaf *Osmanthus* variety, ‘Yinbi Shuanghui,’ Chen et al. found that leaf color is mainly affected by chlorophyll and carotenoid content [65].

**Figure 10:**
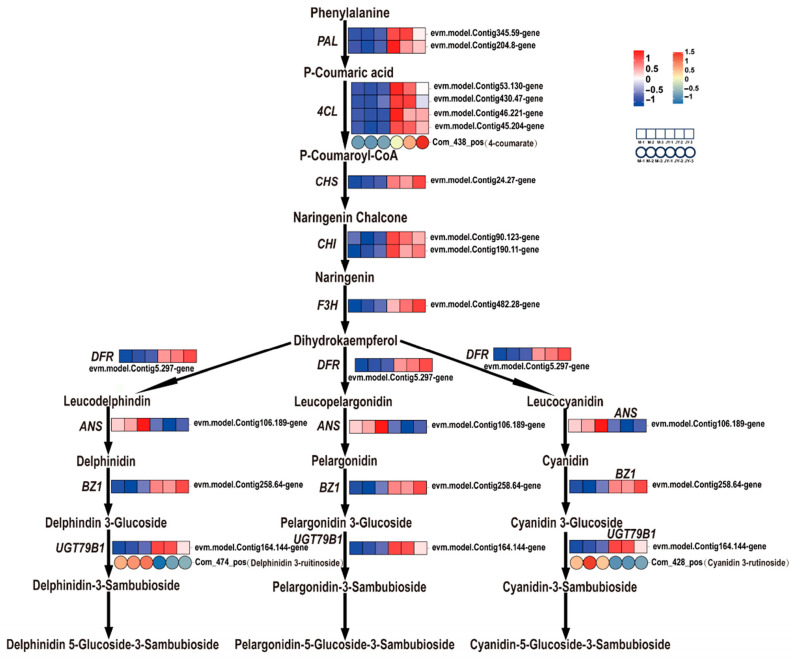
Analysis of differential gene expression associated with anthocyanin biosynthesis. Note: Ruler bars indicate gene/metabolite expression patterns (rectangle: gene, circle: metabolite; red: up-regulated, blue: downregulated, white: no change; normalized signal intensity: −1.0 to 1.0 (gene)/−1.0 to 1.5 (metabolites), consistent with color change from blue to red).

In this study, pigment determinations revealed that, compared to the female parent plant’s leaves, the anthocyanin contents in the yellow and green parts of ‘Jinyu Guihua’ leaves were significantly lower, while there was no significant difference in flavonoid content. This indicates that the change in anthocyanin content affects the phenotype of ‘Jinyu Guihua’ leaves at the physiological level. Further analysis of anthocyanin metabolism showed that there were significant differences in the expression of genes and metabolites between ‘Jinyu Guihua’ and its female parent. A total of 22 DEGs related to the anthocyanin synthesis pathway were identified, including *PAL*, *4CL*, *CHS*, *CHI*, *F3H*, *DFR*, *ANS*, *BZ1*, and *UGT79B1*. Compared to the female parent, *PAL*, *4CL*, *CHS*, *CHI*, *F3H*, *DFR*, *ANS*, *BZ1*, *UGT79B1*, and other related genes were upregulated in ‘Jinyu Guihua’. Among these, 4-coumaroyl CoA ligase (*4CL*) was upregulated, and the content of its corresponding metabolite (4-coumaroyl) was increased. Anthocyanin synthase (*ANS*) was downregulated, and the corresponding metabolites of cyanidin-3-O-rutinoside (Cy3R) and delphinidin-3-O-rutinoside (De3R) were decreased (Figure 10). These results indicate that there is a correlation between the expression of genes and metabolites in the secondary metabolic pathway and leaf development. This finding aligns with research on leaf color transition mechanisms in ‘Zhonghuahongye’. During the shift from purplish-red to brownish-green in ‘Zhonghuahongye’ leaves, metabolomic analysis reveals that significant downregulation of key pigments such as malvidin-3-O-glucoside (Mv3G) and anthocyanins constitutes the core metabolic basis for the color change [66].

In summary, the anthocyanin contents in the yellow and green parts of ‘Jinyu Guihua’ leaves were significantly lower than those in the leaves of the female parent plant. Consistently, *ANS*, a key structural gene in anthocyanin biosynthesis, was significantly downregulated, and the levels of its downstream metabolites, cyanidin-3-O-rutinoside and delphinidin-3-O-rutinoside, were also markedly reduced. The coordinated decrease in *ANS* expression and its corresponding metabolites indicates a strong concordance between transcriptomic and metabolomic changes within the anthocyanin biosynthesis pathway. These results suggest that the reduced accumulation of anthocyanins is closely associated with the suppression of *ANS* at the transcriptional level. Moreover, the decreased levels of cyanidin-3-O-rutinoside and delphinidin-3-O-rutinoside imply that the biosynthetic flux toward colored anthocyanins is limited, which may consequently affect pigment accumulation in the leaves. Taken together, these findings provide mechanistic insight into how altered anthocyanin biosynthesis may contribute to the leaf phenotype variations observed in ‘Jinyu Guihua’.

## 4. Materials and Methods

### 4.1. Plant Materials

The experimental materials were the bud mutation variety, ‘Jinyu Guihua’, and its female parent, which grew under natural conditions in the National *Osmanthus* Germplasm Resources Nursery in Lin’an District, Hangzhou City, Zhejiang Province. Mature leaves of ‘Jinyu Guihua’ and healthy branches of the female parent were selected. Transcriptomic and metabolomic sequencing and fluorescence quantitative PCR experiments were performed using the whole leaves. Paraffin sectioning, transmission electron microscopy, and pigment determination experiments were also conducted. The green and yellow parts of the ‘Jinyu Guihua’ leaf were sampled separately.

### 4.2. Phenotypic Determination

**Figure 11:**
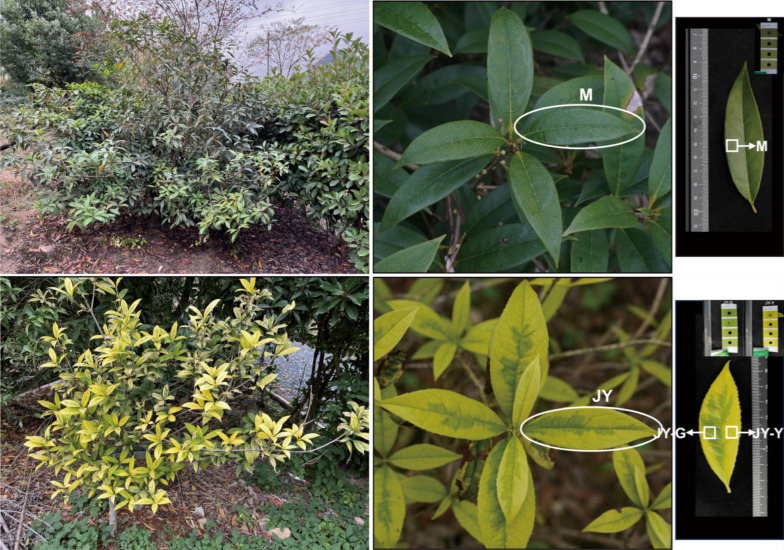
Leaf phenotypes of ‘Jinyu Guihua’ and its maternal parent. Note: M: maternal parent leaf; JY-G: green part of ‘Jinyu Guihua’; JY-Y: Yellow part of ‘Jinyu Guihua’; RHS: Y146A, Y146B, Y151A.

The fresh leaves were collected on a sunny morning, the leaf epidermis was wiped clean, and direct sunlight exposure was subsequently avoided. The leaves’ color was compared against the Royal Horticultural Society Colorimetric Card (RHSCC), and the color code closest to the leaves’ actual color was recorded [67]. The color of the female parent leaf (M) was Y-146A, the green part of the ‘Jinyu Guihua’ leaf (JY-G) was Y-146B, and the yellow part of the ‘Jinyu Guihua’ leaf (JY-Y) was Y-151A (Figure 11).

### 4.3. Experimental Methods for Paraffin Sectioning

We used a slightly modified version of the paraffin sectioning method proposed by Li Heping [68]. Small pieces of roughly 5 mm × 5 mm of the yellow and green parts of the ‘Jinyu Guihua’ leaves (along the main vein and in the middle of the leaf) were selected and placed into FAA fixative, vacuumed, and fixed for at least 24 h. This was followed by dehydration, transparency, waxing, embedding, and slicing (to a thickness of 1.2–1.5 μm). Subsequently, we performed safranin-fast green staining and mounted the tissue sections using neutral gum. Finally, the cells were observed under a microscope, and images were taken and analyzed. The lignified cytoplasm was red, and the cellulose cell wall was green.

### 4.4. Transmission Electron Microscopy Experimental Methods

We used a slightly modified version of the TEM method proposed by Fu Honglan [69]. Small pieces of roughly 1 mm × 1 mm of the yellow and green parts of the ‘Jinyu Guihua’ leaves (along the main vein and in the middle of the leaf) were selected and placed into 4% glutaraldehyde fixative, and washed using 0.1 M PBS (with a pH of 7.4). The prepared 1% osmic acid solution was fixed at room temperature in darkness for 7 h. We then performed ethanol gradient dehydration at room temperature, followed by infiltration of ethanol and the embedding agent. The embedding plates were polymerized in an oven at 60 °C for 48 h, sliced using an ultra-thin slicing machine, and stained with 2% uranyl acetate saturated alcohol solution in the dark. The samples were observed and photographed under a transmission electron microscope.

The number, length, and width of chloroplasts were measured in three randomly selected cells.

### 4.5. Methods of Pigment Content Determination

We used slightly modified versions of the chlorophyll and carotenoid content determination method proposed by Peng [70] and Su [71], and the anthocyanin content determination method proposed by Pietrini [72]. Shaded samples were sealed using 80% acetone (95% ethanol = 1:1 mixed extract). Chlorophyll and carotenoids were extracted at 4 °C for 24 h until the leaves became white. The extracts were then filtered to be used as a reference solution. The optical density (OD) values were measured by spectrophotometry at wavelengths of 440 nm, 645 nm, and 663 nm. Flavonoids and anthocyanins were extracted in darkness using 1% hydrochloric acid ethanol solution for 24 h. The absorbance values were measured via spectrophotometry at wavelengths of 535 nm, 280 nm, and 320 nm. Three biological replicates were selected for all subsequent measurements. The content of each pigment was then calculated using the formula (FW: fresh weight)*.*

### 4.6. RNA Extraction, Library Construction, and Gene Annotation

The total RNA of the three biological replicates was extracted using a TRIzol RNA Purification Kit (Invitrogen, Carlsbad, CA, USA). The quality of the extracted RNA was measured using an Agilent 2100 Bioanalyzer (Agilent Technologies, Palo Alto, CA, USA). A cDNA library was constructed from the qualified RNA samples.

Raw sequencing reads were subjected to quality control using fastp [73] to remove low-quality data and generate clean reads. The filtering procedures included: (1) removal of reads containing adapter sequences; (2) removal of reads with more than 10% ambiguous nucleotides (N); (3) removal of reads composed entirely of adenine (A) bases; and (4) removal of low-quality reads.

High-quality clean readings were then mapped to the reference genome of *Osmanthus fragrans* [74]. An analysis of the differential RNA expression was performed between the two different groups (as well as the edgeR between the two samples [75]) using DESeq2. Differential gene screening was performed using an FDR threshold of 1, and the gene function was annotated using KEGG and GO.

### 4.7. HPLC MS/MS Methods

The samples were vacuum freeze-dried and ground to a powder at 1.5 Hz for 400 min with a grinding machine (MM30, Retsch, Haan, Germany). An amount of 100 mg of the powder was dissolved in 1 mL of extract and refrigerated overnight at 4 °C. The sample was vortexed three times during the refrigeration period. Following centrifugation (at 10,000 *g* for 10 min), the supernatant was extracted and filtered through a microporous membrane (with a pore size of 0.22 μm). Analyst 1.6.1 was used to calculate the area of each chromatographic peak and align the peaks of different samples according to their spectral patterns and retention times. The obtained *m*/*z* values, RT, and fragment patterns were compared against the standard. Finally, the data matrices of the retention times, mass-to-charge ratios, and peak intensities were obtained, and the metabolites were determined using a custom GENE DENOVO database.

The VIP value of the multivariate statistical analysis (OPLS-DA) and the *p* value of the univariate statistical analysis (*t*-test) were combined to screen the significant differential metabolites between different comparison groups [76]. The threshold of significant difference was a VIP value of ≥1 in the OPLS-DA model and a *t*-test *p*-value of <0.05. Based on the qualitative results, the corresponding Compound IDs (C_id) were found in the KEGG database [77]. The pathway criterion for significant enrichment in the differentially expressed metabolites was a Q value of ≤0.05.

### 4.8. qRT-PCR Methods

The total RNA was extracted using the Omega Plant RNA Kit (Omega, New York, NY, USA), and the cDNA was synthesized using a cDNA synthesis kit (HiScript II 1st Strand cDNA Synthesis Kit, Novozymes Biotech Co., Ltd., Tianjin, China) according to the manufacturer’s instructions. The instrument used was a Nanodrop One Microvolume UV-Vis Spectrophotometer (Thermo Fisher, Waltham, MA, USA) with a reaction system of 20 μm. The primer sets of the target and reference genes are listed in Table A1 (Appendix A). The amplification reaction included an initial denaturation step at 95 °C for 30 s, followed by 40 cycles at 95 °C for 10 s and 55 °C for 30 s. The relative expression of the selected genes was calculated using 2^−^^ΔΔCt^. Each gene had three biological replicates.

### 4.9. Data Analysis

The measurement results were statistically analyzed using Excel (2019), SPSS (26), and Origin (2021) with an FDR of <0.05. An orthogonal partial least squares discriminant analysis (OPLS-DA) was performed using R 4.0, and cross-validation was performed using the established model. Metabolites with significant differences (i.e., with a *t*-test *p* of <0.05 and a VIP of >1) were screened out. After screening out the differential genes and metabolites, the KEGG database was used to conduct enrichment and trend analyses to evaluate the obtained data through principal component analysis and *t*-testing. Gene ontology (GO), Kyoto Encyclopedia of Genes and Genomes (KEGG) pathway, and differential genes and metabolites analyses were performed using the free online data analysis tool on the OmicShare platform (http://www.omicshare.com/tools, accessed on 16 November 2025).

## 5. Conclusions

**Figure 12:**
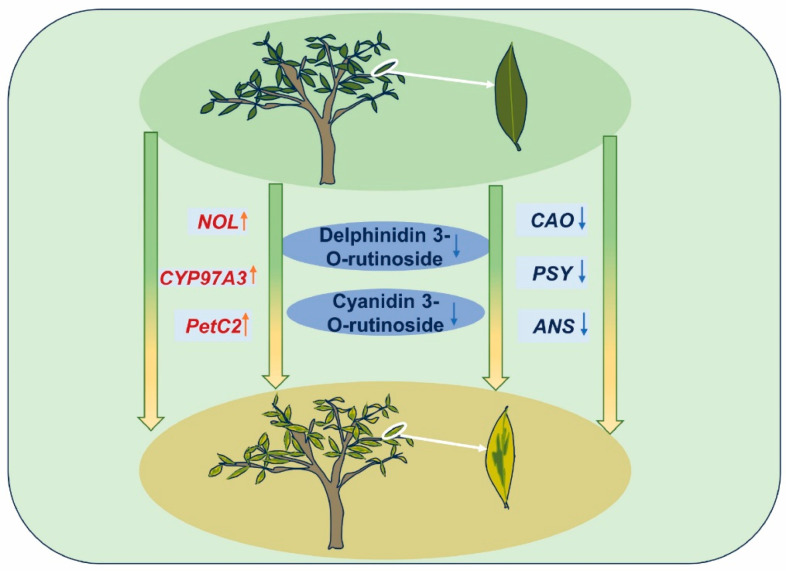
Pattern diagram of the color rendering mechanism of ‘Jinyu Guihua’. Note: red: upward, blue: downward.

In summary, the combined results of physiological experiments, transcriptomics, and metabolomics in this study preliminarily clarify a mechanistic model of the leaf color variations seen in ‘Jinyu Guihua’ (Figure 12). We speculate that the abnormal chlorophyll cycle (*CAO* downregulation and *NOL* upregulation) and abnormal chloroplast structure (matrix thylakoid expansion, plastid globule proliferation) in the chlorophyll metabolism pathway are closely associated with the leaf color variations, particularly in the yellow sectors of ‘Jinyu Guihua’ leaves. In terms of carotenoid metabolism, the abnormal conversion to the β-carotene branch (*CYP97A3* upregulation and *VDE* upregulation) leads to an increase in yellow zeaxanthin content. In addition, abnormal anthocyanin metabolism (*ANS* downregulation, Cy3R downregulation, and De3R downregulation) leads to anthocyanins not affecting the plant’s leaf color. As a result, the leaf color is mainly affected by the presence of chlorophyll and carotenoids. Taken together, these factors likely contribute to the leaf color variations seen in ‘Jinyu Guihua’.

This study provides a solid theoretical foundation for the further utilization and improvement of the cultivar ‘Jinyu Guihua’ by identifying key candidate genes associated with its leaf color variation. These findings offer valuable molecular targets for future functional studies and germplasm innovation. Moreover, the results serve as an important reference for investigating leaf color mutants in other ornamental plants, thereby contributing to a deeper understanding of pigment-related regulatory mechanisms and supporting the conservation and utilization of plant biodiversity.

## Data Availability

All data generated or analyzed during this study are included in this published article, its Appendix A, and publicly available repositories. The raw RNA-Seq reads are available at the NCBI Sequence Read Archive (SRA): PRJNA1081802.

## Appendix A

**Table A1:** Real-time fluorescence quantitative PCR primers.

Gene ID	Sense Primer	Anti-Sense Primer
*Actin*	CGTGGCACTTGACTATGAA	TCTGGGCAACGGAATCTCT
evm.model.Contig164.99-gene	ATGGGCTGTTTGCTCCTACC	AGCACCCAAAAGGAGCAAGT
evm.model.Contig26.218-gene	AAGCTTGCCTCGAAGGAGTC	CGTGGACTTCCCAGTTCCTC
evm.model.Contig78.196-gene	CTCCGGGGATTAGTCTGGGA	TTCGAGGCAAGCTTTTGTGC
evm.model.Contig79.172-gene	AGTTTGATCAGCCCTCGGTT	TCTCGTGGAGTTCGCATGAC
evm.model.Contig72.79-gene	AGCATTGGATCGTTGGGAGG	ATTTCCAGAGGTCCATCCGC
evm.model.Contig240.94-gene	TCGGAGCACCTCCTGTAGAA	CGCAGTTGGCGTCTGTAATG
evm.model.Contig9.158-gene	ACCCATACATGCCTTTGCCA	CAGGCGCTTCACGATAGAGT
evm.model.Contig204.8-gene	TGGTACAGCAGTTGGCTCTG	CAATTTGGCCAGGATGGTGC
evm.model.Contig24.27-gene	GCCGACTACCAACTCACCAA	CCCGGAATGTAACCGCAGTA
evm.model.Contig190.11-gene	GCTAGAGAGCGCGGTAAGAG	TGCAAATGCAATCTCAGCGG
evm.model.Contig114.161-gene	TGCCAGCAGTAGGGACTTTG	GGCCTGCTAACATGTGGTCT
evm.model.Contig182.127-gene	TTTTTCCAAGAAGCGCCACG	GGGTACGTGATCGTCCTTCC
